# miR-132-3p Modulates DUSP9-Dependent p38/JNK Signaling Pathways to Enhance Inflammation in the Amnion Leading to Labor

**DOI:** 10.3390/ijms23031864

**Published:** 2022-02-07

**Authors:** Zhuxia Zhong, Zezhang Liu, Rong Zheng, Jin Chai, Siwen Jiang

**Affiliations:** Agricultural Ministry Key Laboratory of Swine Breeding and Genetics & Key Laboratory of Agricultural Animal Genetics, Breeding and Reproduction of Ministry of Education, Huazhong Agricultural University, Wuhan 430070, China; zzx05031319@163.com (Z.Z.); zzl19920507@hotmail.com (Z.L.); zhengrong@mail.hzau.edu.cn (R.Z.); chaijin@mail.hzau.edu.cn (J.C.)

**Keywords:** miR-132-3p, *DUSP9*, p38/JNK, labor, inflammation, amnion

## Abstract

Labor is a process of inflammation and hormonal changes involving both fetal and maternal compartments. MicroRNA-132-3p (miR-132-3p) has been reported to be involved in the development of inflammation-related diseases. However, little is known about its potential role in labor onset. This study aimed to explore the mechanism of miR-132-3p in amnion for labor initiation. In the mouse amnion membranes, the expression of miR-132-3p was found to increase gradually during late gestation. In human amniotic epithelial cell line (WISH), upregulation of miR-132-3p was found to increase proinflammatory cytokines and cyclooxygenase 2 (COX2) as well as prostaglandin E2 (PGE2), which was suppressed by miR-132-3p inhibitor. Dual-specificity phosphatase 9 (DUSP9) was identified as a novel target gene of miR-132-3p, which could be negatively regulated by miR-132-3p. DUSP9 was present in the mouse amnion epithelial cells, with a decrease in its abundance at 18.5 days post coitum (dpc) relative to 15.5 dpc. Silencing *DUSP9* was found to facilitate the expression of proinflammatory cytokines and COX2 as well as PGE2 secretion in WISH cells, which could be attenuated by p38 inhibitor SB203580 or JNK inhibitor SP600125. Additionally, intraperitoneal injection of pregnant mice with miR-132-3p agomir not only caused preterm birth, but also promoted the abundance of COX2 as well as phosphorylated JNK and p38 levels, and decreased DUSP9 level in mouse amnion membranes. Collectively, miR-132-3p might participate in inflammation and PGE2 release via targeting *DUSP9*-dependent p38 and JNK signaling pathways to cause preterm birth.

## 1. Introduction

Labor or parturition, an event indicating the end of pregnancy, is a complex physiological process involving interactions between the myometrium and signals derived from fetus, fetal membranes, and placenta [1]. Fetal membranes provide mechanical support to protect the growing fetus as well as delivery signals to inform the mother of fetal maturation through endocrine or paracrine pathways to ensure the smooth delivery of fetus [2,3]. There is substantial evidence that indicates that fetal membrane-derived signals, including prostaglandins (PGs) and inflammation, are involved in the initiation and progression of parturition [4,5,6,7]. Specifically, prostaglandins and inflammation were reported to induce changes in cervical structure, contraction of the uterus, and weakening of fetal membranes at the onset of labor [8]. Excessive or premature activation of these signals might cause preterm labor, resulting in maternal and neonatal pathological damages and diseases [9]. Therefore, understanding the molecular mechanism of prostaglandins and inflammation in fetal membranes during labor may facilitate the prediction or prevention of preterm birth.

MiRNAs, a class of noncoding small RNAs, play an essential role in parturition initiation. For instance, the expression of miR-181a could be suppressed by E2 in myometrium near term, upregulating proinflammatory cytokines and leading to labor [10]. MiR-200, miR-200a, miR-199a/miR-214 cluster, and miR-212-3p have been shown to be related to the uterine activity from quiescent to contractile during pregnancy and labor [11,12,13,14]. In our previous study, we have observed an increase of miR-132-3p expression and a corresponding increase of COX2 expression in the fetal membranes of pregnant large white pigs at day 114 and 112 of pregnancy with or without signs of labor onset [15]. MiR-132-3p has been extensively explored in the fields of oncology, immunology, and neurology [16,17]. For instance, miR-132-3p was found to act as an anti-inflammatory regulator of the brain to the body by targeting acetylcholinesterase [18]. A recent study indicated that miR-132-3p could exacerbate cisplatin-induced acute kidney injury by upregulating inflammatory and apoptosis pathways [19]. Other studies showed that miR-132-3p participated in the pathogenesis of gestational diabetes mellitus and preeclampsia through the regulation of viability and invasiveness in trophoblast cells [20,21]. However, to our knowledge, no study has ever been performed on the role of miR-132-3p in labor.

Dual-specificity phosphatase 9 (DUSP9), which is also named MAP kinase phosphatase-4 (MKP-4) and a member of the dual-specificity phosphatase superfamily, can dephosphorylate serine/threonine or tyrosine residues to decrease the activity of mitogen-activated protein kinases (MAPK), including ERK1/2, JNK, and p38 [22,23,24]. In recent studies, *DUSP9* has been revealed to participate in inflammatory responses in the development of nonalcoholic fatty liver disease (NAFLD) and nonalcoholic steatohepatitis (NASH) via p38 and JNK signaling pathways [25]. *DUSP9* is dispensable for mammalian embryonic development, but essential for placental function, and its deletion can cause embryonic death [26]. Moreover, *DUSP9* was reported to be associated with pregnancy complications, such as gestational diabetes mellitus and preeclampsia [27,28]. However, little information is available about the roles of *DUSP9* in the process of parturition.

In the current study, by predicting miR-132-3p binds to *DUSP9*, we aimed to investigate how miR-132-3p and its target *DUSP9* participate in the regulation of inflammation and prostaglandins for parturition initiation by analyzing their interactions using human amnion epithelial cells and a mouse model. Our results will not only enrich insights into the roles of miRNAs in amnion for the onset of labor, but also may provide a target for the diagnosis and treatment of preterm birth.

## 2. Results

### 2.1. miR-132-3p Was Upregulated in Mouse Amnion Membranes during Late Gestation

**Figure 1:**
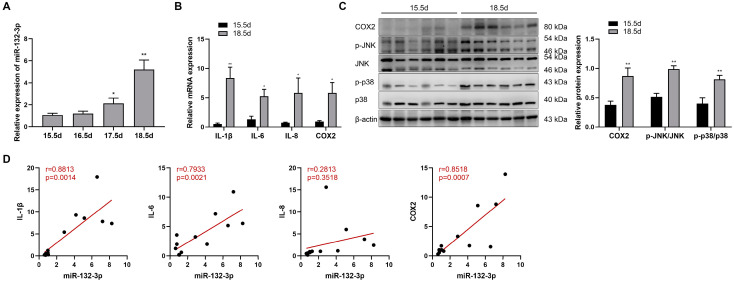
Upregulated expressions of miR-132-3p and labor-associated genes are observed in mouse amnion membranes during late gestation. (**A**) RT-qPCR analysis of miR-132-3p level in the amnion membranes from pregnant mice at 15.5–18.5 days post-coitum (dpc) (*n* = 6–8). (**B**) RT-qPCR analysis of the mRNA expression of *IL-1β*, *IL-6*, *IL-8*, and *COX2* in mouse amnion membranes at 15.5 and 18.5 dpc (*n* = 6–8). (**C**) Western blot analysis of the protein expression of COX2, JNK, and p38 as well as the phosphorylated JNK, and p38 levels in mouse amnion membranes at 15.5 and 18.5 dpc using β-actin as internal control (*n* = 6). (**D**) Gene expression correlation analysis between miR-132-3p and *IL-1β*, *IL-6*, *IL-8*, and *COX2* mRNA in mouse amnion membranes at 15.5 dpc (*n* = 6–7) and 18.5 dpc (*n* = 6). Pearson correlation test and values of r and *p* are shown for each analysis. Data were shown as mean ± SEM. * *p* < 0.05, ** *p* < 0.01 vs. 15.5 dpc.

The potential role of miR-132-3p in labor was explored by analyzing the expression of miR-132-3p in mouse amnion membranes during late gestation. In Figure 1A, miR-132-3p showed a gradual increase from 15.5 to 18.5 dpc in mouse amnion membranes, coupled with significant upregulation in the mRNA expression of *IL-1β*, *IL-6*, *IL-8*, and *COX2* at 18.5 dpc relative to 15.5 dpc (Figure 1B). Additionally, Western blot analysis revealed a significant rise in COX2 protein as well as phosphorylated p38 and JNK levels in mouse amnion membranes at 18.5 dpc versus 15.5 dpc (Figure 1C). Furthermore, the expression of miR-132-3p in mouse amnion membranes were positively correlated with that of *IL-1β*, *IL-6*, and *COX2* mRNA, but not in the expression of miR-132-3p and *IL-8* mRNA (Figure 1D), suggesting a potential role of amniotic miR-132-3p in the process of parturition.

### 2.2. miR-132-3p Increased the Abundance of Proinflammatory Cytokines and PGE2 in WISH Cells

**Figure 2:**
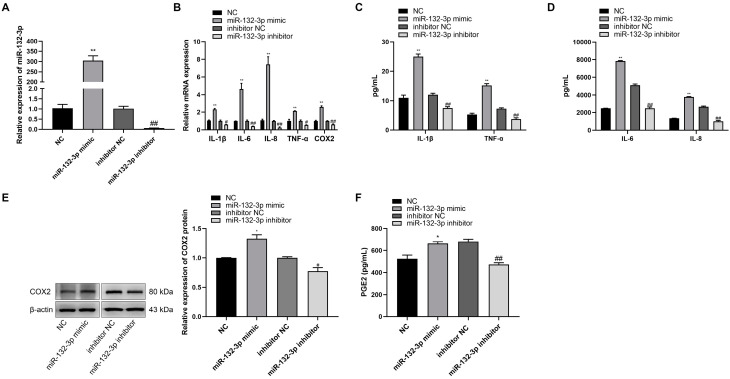
miR-132-3p promotes the expression of proinflammatory cytokines and COX2 as well as PGE2 in WISH cells. WISH cells were transfected with miR-132-3p mimic, miR-132-3p inhibitor, or the corresponding controls. (**A**) RT-qPCR analysis of miR-132-3p expression in WISH cells. (**B**) RT-qPCR analysis of the expression of *IL-1β*, *IL-6*, *IL-8*, *TNF-α*, and *COX2* in WISH cells. Magnetic Luminex Assays of the secretion of IL-1β, TNF-α (**C**), IL-6, and IL-8 (**D**) in WISH cells. (**E**) Western blot analysis of COX2 level in WISH cells. (**F**) ELISA analysis of PGE2 secretion in WISH cells. Data were shown as mean ± SEM, *n* = 3. * *p* < 0.05, ** *p* < 0.01 vs. NC. # *p* < 0.05, ## *p* < 0.01 vs. inhibitor NC.

The role of miR-132-3p on WISH cells was investigated by transfection of the cells with miR-132-3p mimic, miR-132-3p inhibitor, or their respective controls. The expression of miR-132-3p showed a significant increase in miR-132-3p mimic relative to the control mimic, but was significantly suppressed by miR-132-3p inhibitor (Figure 2A). RT-qPCR analysis showed that the levels of *IL-1β*, *IL-6*, *IL-8*, *TNF-α*, and *COX2* were significantly higher in miR-132-3p mimic-treated WISH cells, but lower in the miR-132-3p inhibitor-treated WISH cells when compared with their respective controls (Figure 2B). Magnetic Luminex Assays showed that the concentrations of IL-1β, IL-6, IL-8, and TNF-α in the culture mediums were significantly higher in the miR-132-3p mimic group, but lower in its inhibition group relative to their respective controls (Figure 2C,D). Western blot analysis showed that the expression of COX2 protein had an increase in the miR-132-3p mimic group, but a decrease in the miR-132-3p inhibitor group as compared with their respective controls (Figure 2E). ELISA analysis revealed a higher concentration of PGE2 in miR-132-3p mimic-treated WISH cells, but a lower concentration in miR-132-3p inhibitor-treated cells relative to their respective controls (Figure 2F). These results confirmed that overexpression of miR-132-3p could promote the release of proinflammatory cytokines and PGE2.

### 2.3. miR-132-3p Directly Targets DUSP9

**Figure 3:**
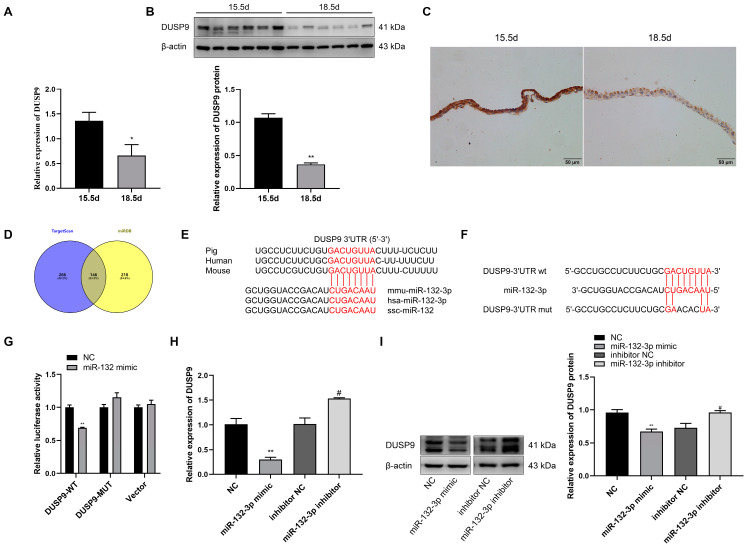
*DUSP9* is a target gene of miR-132-3p. RT-qPCR (**A**) and Western blot (**B**) analysis of DUSP9 level in mouse amnion membranes at 15.5 and 18.5 dpc (*n* = 6–8). (**C**) Immunohistochemical staining for DUSP9 in the amnion membranes from pregnant mice at 15.5 or 18.5 dpc. Nuclei are stained with hematoxylin. Scale bar, 50 μm. (**D**) Venn diagram for the overlap of target genes predicted by TargetScan and miRDB. (**E**) Conservation of the miR-132-3p target sequence in *DUSP9* 3′UTR and mature miR-132-3p sequence among different species. (**F**) MiR-132-3p binding site in *DUSP9* 3′UTR predicted by TargetScan. (**G**) Luciferase reporter assay of WISH cells co-transfected with miR-132-3p mimic or NC and pmirGLO-*DUSP9*-3′UTR WT, pmirGLO-*DUSP9*-3′UTR MUT, or empty vector. RT-qPCR (**H**) and Western blot (**I**) analysis of the expression of DUSP9 in WISH cells transfected with miR-132-3p mimic, NC, miR-132-3p inhibitor, or inhibitor NC. Data were shown as mean ± SEM, *n* = 3. * *p* < 0.05, ** *p* < 0.01 vs. NC. # *p* < 0.05 vs. inhibitor NC.

Figure 3D shows the potential target genes of miR-132-3p predicted by TargetScan and miRDB, with *DUSP9* as one of the many target genes. A recent study revealed that *DUSP9* knockout could induce the production of liver inflammation [25]. RT-qPCR and Western blot results showed a significant decrease in the abundance of *DUSP9* mRNA and protein in mouse amnion membranes at 18.5 dpc relative to 15.5 dpc (Figure 3A,B). Similarly, immunohistochemical staining of mouse amnion membranes revealed that DUSP9 in amnion epithelial cells showed weaker signal intensity at 18.5 dpc than at 15.5 dpc (Figure 3C). Sequence alignment indicated the mature sequence of miR-132-3p and the binding site of miR-132-3p in the *DUSP9* 3′UTR are highly conserved across species (Figure 3E). Whether miR-132-3p directly targets *DUSP9* 3′UTR was verified by co-transfecting WISH cells with miR-132-3p mimic and pmirGLO-*DUSP9*-3′UTR WT construct, resulting in a significant downregulation of the luciferase activity (Figure 3F,G). However, miR-132-3p mimic had no effect on the luciferase activity in WISH cells transfected with pmirGLO empty vector or pmirGLO-*DUSP9*-3′UTR MUT construct, where four bases containing the miR-132-3p binding site were muted (Figure 3F,G). Compared with their respective controls, the *DUSP9* expression was significantly suppressed in miR-132-3p mimic-treated WISH cells, but enhanced in the miR-132-3p inhibitor-treated cells (Figure 3H). Moreover, DUSP9 protein expression in WISH cells was suppressed by overexpression of miR-132-3p, but enhanced by inhibition of miR-132-3p (Figure 3I). Taken together, *DUSP9* was the direct target gene of miR-132-3p and could be negatively regulated by miR-132-3p.

### 2.4. Inhibition of DUSP9 Promoted Inflammatory Responses and PGE2 Secretion in WISH Cells

**Figure 4:**
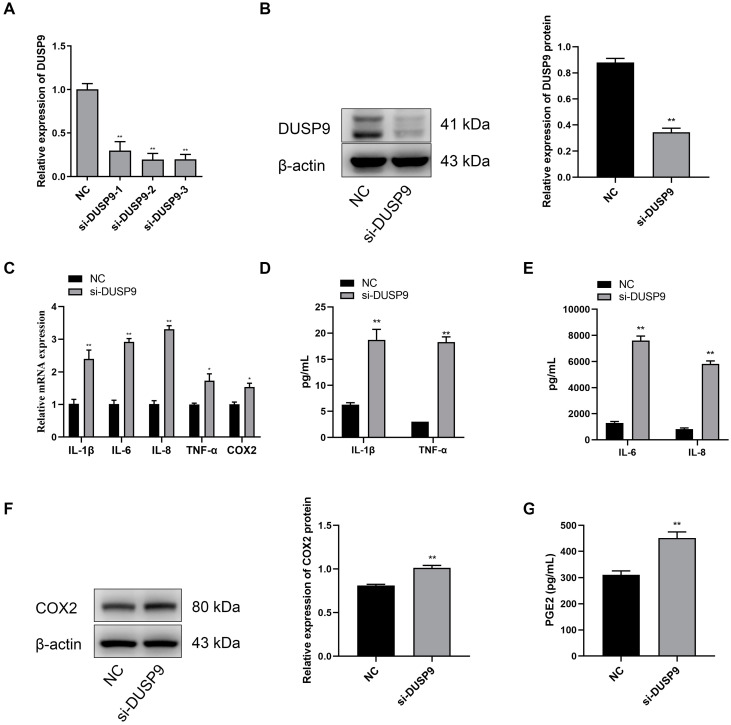
Inhibition of *DUSP9* promotes proinflammatory cytokines and COX2 as well as PGE2 expression in WISH cells. WISH cells were transfected with *DUSP9* siRNA fragment and negative control, respectively. (**A**) RT-qPCR of *DUSP9* expression. (**B**) Western blot analysis of DUSP9 protein level. (**C**) RT-qPCR analysis of the expression of *IL-1β*, *IL-6*, *IL-8*, *TNF-α*, and *COX2*. Magnetic Luminex Assays of the concentrations of IL-1β, TNF-α (**D**), IL-6, and IL-8 (**E**). (**F**) Western blot analysis of the protein expression of COX2. (**G**) ELISA analysis of PGE2 secretion level. Data were shown as mean ± SEM, *n* = 3. * *p* < 0.05, ** *p* < 0.01 vs. NC.

Compared with the negative control, the WISH cells transfected with *DUSP9* interference fragments showed significant reduction in the mRNA and protein expression levels of DUSP9 (Figure 4A,B). Meanwhile, RT-qPCR analysis showed that the treatment of *DUSP9* siRNA contributed to the expression of *IL-1β*, *IL-6*, *IL-8*, *TNF-α*, and *COX2* in WISH cells (Figure 4C). Consistently, Magnetic Luminex Assays indicated that WISH cells treated with *DUSP9* siRNA showed a significant increase in the concentrations of IL-1β, IL-6, IL-8, and TNF-α (Figure 4D,E). Western blot analysis confirmed the increase of COX2 protein in WISH cells transfected with *DUSP9* siRNA (Figure 4F). Moreover, the ELISA results showed a significant increase of PGE2 secretion in *DUSP9* siRNA-treated WISH cells (Figure 4G). These findings indicated that *DUSP9* knockdown could promote inflammatory responses and PGE2 secretion.

### 2.5. miR-132-3p Induced Inflammation and PGE2 via DUSP9 in WISH Cells

**Figure 5:**
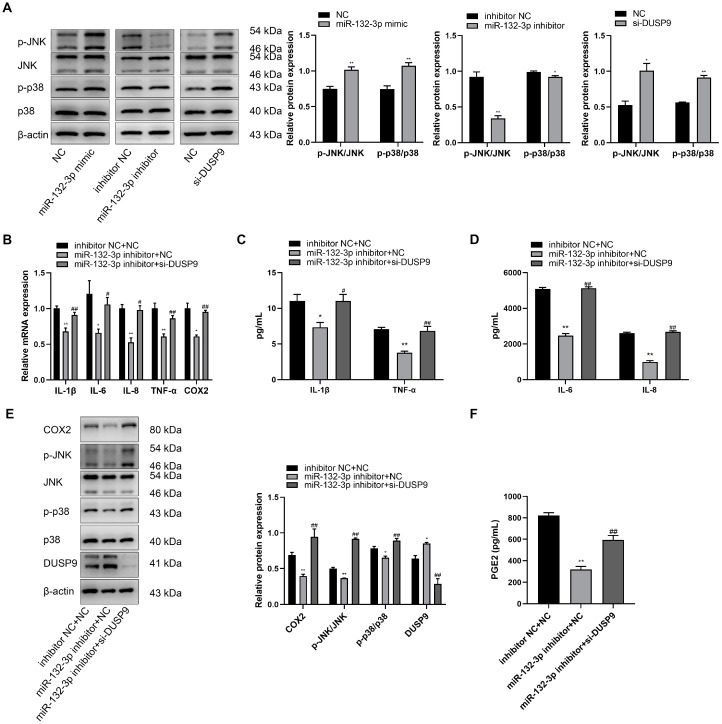
*DUSP9* knockdown attenuates the anti-inflammatory effects of miR-132-3p loss in WISH cells. (**A**) Western blot analysis of the protein expression level of JNK and p38 as well as the phosphorylated JNK and p38 levels in WISH cells transfected with miR-132-3p mimic, miR-132-3p inhibitor, si-*DUSP9*, or the corresponding controls. * *p* < 0.05, ** *p* < 0.01 vs. the corresponding controls. (**B**) RT-qPCR analysis of the expression of *IL-1β*, *IL-6*, *IL-8*, *TNF-α*, and *COX2* in WISH cells transfected with both inhibitor NC and NC, both miR-132-3p inhibitor and NC, or both miR-132-3p inhibitor and si-*DUSP9*. Magnetic Luminex Assays of the concentrations of IL-1β, TNF-α (**C**), IL-6, and IL-8 (**D**) in WISH cells transfected with both inhibitor NC and NC, both miR-132-3p inhibitor and NC, or both miR-132-3p inhibitor and si-*DUSP9*. (**E**) Western blot analysis of the protein expression level of COX2, JNK, p38, and DUSP9 as well as the phosphorylated JNK and p38 levels in WISH cells transfected with both inhibitor NC and NC, both miR-132-3p inhibitor and NC, or both miR-132-3p inhibitor and si-*DUSP9*. (**F**) ELISA analysis of PGE2 secretion in WISH cells transfected with both inhibitor NC and NC, both miR-132-3p inhibitor and NC, or both miR-132-3p inhibitor and si-*DUSP9*. Data were shown as mean ± SEM, *n* = 3. * *p* < 0.05, ** *p* < 0.01 vs. inhibitor NC + NC, # *p* < 0.05, ## *p* < 0.01 vs. miR-132-3p inhibitor + NC.

It has been reported that DUSP9 can inactivate p38 and JNK pathways by dephosphorylating serine/threonine and tyrosine residues [22]. As shown in Figure 5A, the protein levels of phosphorylated p38 and JNK were obviously increased when the cells were transfected with miR-132 mimic or *DUSP9* siRNA compared to NC, but downregulated by the treatment of miR-132-3p inhibitor relative to inhibitor NC. To explore whether miR-132-3p induces inflammation and PGE2 expression by DUSP9 downregulation, WISH cells were co-transfected with miR-132-3p inhibitor or inhibitor NC and si-*DUSP9* or NC. RT-qPCR analysis revealed a marked decrease in the mRNA levels of *IL-1β*, *IL-6*, *IL-8*, *TNF-α*, and *COX2* due to inhibition of miR-132-3p, which was recovered by co-transfection of *DUSP9* siRNA and miR-132-3p inhibitor (Figure 5B). Magnetic Luminex Assays showed lower concentrations of IL-1β, IL-6, IL-8, and TNF-α for miR-132-3p inhibitor treatment relative to the control, but this effect was rescued by *DUSP9* siRNA treatment (Figure 5C,D). Western blot analysis showed that inhibition of miR-132-3p remarkably increased the DUSP9 protein expression, and suppressed the COX2 protein level as well as phosphorylated JNK and p38 levels, which could be mitigated by knockdown of *DUSP9* in miR-132-3p inhibitor-treated WISH cells (Figure 5E). Moreover, ELISA results showed that miR-132-3p inhibitor obviously repressed the concentration of PGE2 in the supernatant, which was reversed by *DUSP9* siRNA transfection (Figure 5F). In summary, miR-132-3p could promote the expression of proinflammatory cytokines, COX2, and PGE2 as well as the activation of p38 and JNK signaling pathways via targeting *DUSP9*.

### 2.6. Silencing DUSP9-Induced Inflammation and COX2 as Well as PGE2 Depended on Activation of p38 and JNK Signaling Pathways in WISH Cells

**Figure 6:**
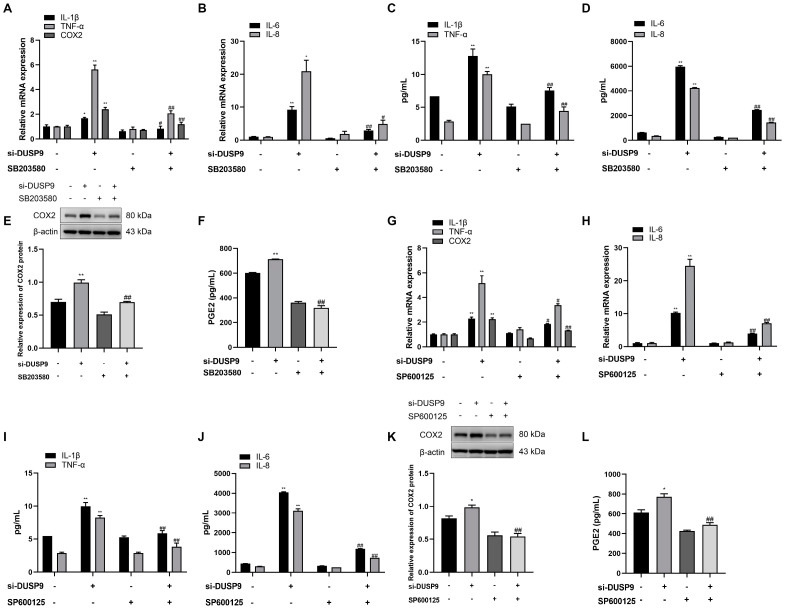
The role of p38 and JNK in *DUSP9* siRNA-induced expression of proinflammatory cytokines and COX2 as well as PGE2 in WISH cells. WISH cells were transfected with si-*DUSP9* or NC for 24 h, followed by 24 h of treatment with DMSO, p38 inhibitor SB203580 (10 μM), or JNK inhibitor SP600125 (20 μM). RT-qPCR analysis of the expression of *IL-1β*, *TNF-α*, *COX2* (**A**,**G**), *IL-6*, and *IL-8* (**B**,**H**). Magnetic Luminex Assays of the secretion of IL-1β, TNF-α (**C**,**I**), IL-6, and IL-8 (**D**,**J**). Western blot analysis of COX2 expression (**E**,**K**). ELISA analysis of PGE2 level (**F**,**L**). Data were shown as mean ± SEM, *n* = 3. * *p* < 0.05, ** *p* < 0.01 vs. control (0), # *p* < 0.05, ## *p* < 0.01 vs. si-*DUSP9*.

Whether *DUSP9* is involved in the process of inflammatory responses and COX2 as well as PGE2 production through p38 and JNK signaling pathways was investigated by treating WISH cells with *DUSP9* siRNA in the presence or absence of DMSO, p38 inhibitor SB203580 (10 μM), and JNK inhibitor SP600125 (20 μM). RT-qPCR analysis indicated that treatment with SB203580 and SP600125 could significantly attenuate *DUSP9* siRNA-induced increases in the mRNA levels of *IL-1β*, *IL-6*, *IL-8*, *TNF-α*, and *COX2* (Figure 6A,B,G,H). Meanwhile, Magnetic Luminex Assays demonstrated that induction of IL-1β, IL-6, IL-8, and TNF-α by *DUSP9* siRNA was significantly attenuated by the treatment of SB203580 and SP600125 (Figure 6C,D,I,J). Moreover, Western blot and ELISA analysis displayed that SB203580 and SP600125 could significantly block *DUSP9* siRNA-induced increases in COX2 protein expression and PGE2 secretion (Figure 6E,F,K,L). These results suggest that *DUSP9* regulated the expression of IL-1β, IL-6, IL-8, TNF-α, and COX2 as well as PGE2 release through p38 and JNK signaling pathways.

### 2.7. Overexpression of miR-132-3p Caused Preterm Labor in Mice

**Figure 7:**
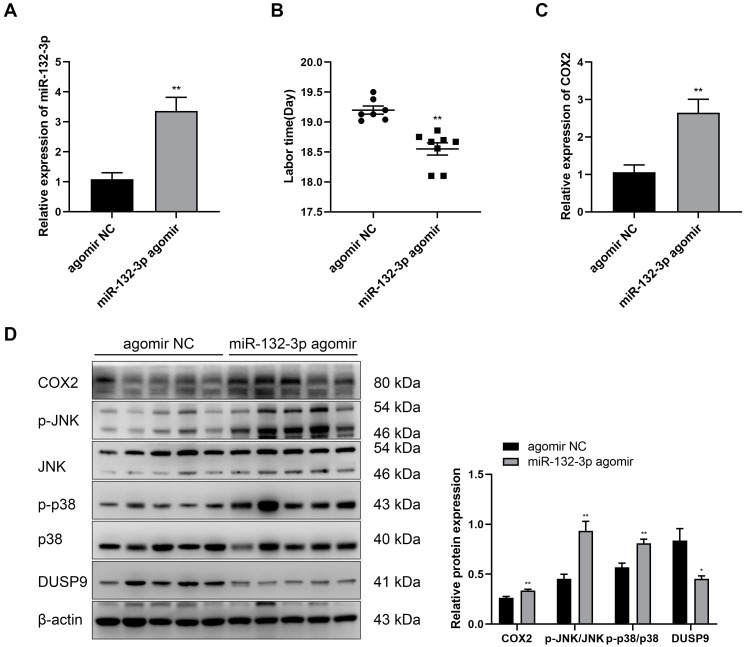
Overexpression of miR-132-3p causes preterm labor in mice. At day 15.5 of pregnancy, the pregnant mice were intraperitoneally injected with miR-132-3p agomir or agomir NC. (**A**) RT-qPCR analysis of the expression of miR-132-3p in mouse amnion membranes (*n* = 5). (**B**) Labor time of pregnant mice (*n* = 7–8). (**C**) RT-qPCR analysis of *COX2* expression in mouse amnion membranes (*n* = 5). (**D**) Western blot analysis of the protein levels of COX2, JNK, p38, and DUSP9 as well as the phosphorylated JNK and p38 levels in mouse amnion membranes (*n* = 5). Data were shown as mean ± SEM. * *p* < 0.05, ** *p* < 0.01 vs. agomir NC.

The function of miR-132-3p during pregnancy and labor was further investigated through intraperitoneal injection of pregnant mice with agomir NC or miR-132-3p agomir at day 15.5 of pregnancy. RT-qPCR results revealed a significant increase in the expression of miR-132-3p in the amnion membranes of mice injected miR-132-3p agomir (Figure 7A). miR-132-3p agomir injection significantly shortened the gestational days of pregnant mice and caused preterm birth at 18.55 ± 0.10 dpc relative to the term birth at 19.20 ± 0.07 dpc for the agomir NC injected mice, without causing any maternal and fetal death (Figure 7B). Additionally, RT-qPCR analysis showed a marked increase in the *COX2* level in the amnion membranes of mice receiving miR-132-3p agomir (Figure 7C). Western blot analysis found a significant decrease in DUSP9 level and upregulation in the protein level of COX2 as well as phosphorylated p38 and JNK levels in mouse amnion membranes upon miR-132-3p agomir injection (Figure 7D). These results confirmed a critical role of miR-132-3p in labor initiation by targeting the DUSP9-p38/JNK axis in vivo.

## 3. Discussion

Labor is a process of inflammation [29], and inflammation, which is recognized as the infiltration of immune cells and the production of proinflammatory cytokines, occurs in the cervix, myometrium, and fetal membranes, facilitating cervical remodeling, uterine contractility, and membrane rupture in both term and preterm labor [30,31]. Our results indicated that miR-132-3p expression was significantly increased in mouse amnion membranes during late gestation. Overexpression of miR-132-3p was shown to increase the expression of labor-associated inflammatory cytokines, including IL-1β, IL-6, IL-8, and TNF-α. Previous studies have reported the involvement of miR-132-3p in various diseases. MiR-132-3p was decreased in serum and placenta tissues of patients with gestational diabetes mellitus (GDM) and acts a protective effect on GDM by promoting trophoblast cell proliferation [20]. miR-132-3p was also shown to be involved in the development of preeclampsia by promoting trophoblast cells viability and invasiveness, and inhibiting apoptosis via targeting *DAPK-1* [21]. There is growing body of evidence indicating that the activation of ERK, p38, JNK, NF-κB, and STAT3 signaling pathways participate in the process of initiation parturition and preterm labor, particularly in inflammatory and hormonal aspects [32,33,34,35]. The actions of miR-132-3p are closely related to these signaling pathways. For instance, TGF-β-induced miR-132-3p expression could shift from inflammatory to the proliferative phase to accelerate skin wound healing through modulating HBEGF-dependent NF-κB, ERK, and STAT3 signaling pathways [36]. Resveratrol and Notoginsenoside R1-induced miR-132-3p expression were reported to attenuate LPS-induced inflammation by blocking the activation of p38 and JNK signaling pathways in PC-12 cells [37,38]. Additionally, Fang et al. [39] showed that overexpression of miR-132-3p suppressed the macrophage M1 inflammation by targeting *MEKK3* to inactivate NF-κB and p38/JNK signaling pathways, ultimately attenuating spinal cord ischemia-reperfusion (SCIR) injury in rat. On the contrary, Diao et al. [40] reported that cigarette smoke extract (CSE) induced miR-132-3p expression and suppressed the expression suppressor of cytokine signaling 5 (SOCS5), promoting the levels of inflammatory cytokines in THP-1 and BEAS-2B cells. These studies indicate the involvement of miR-132-3p in numerous biological processes, exerting anti-inflammatory or pro-inflammatory effects upon targeting various genes to regulate different signaling pathways. In our previous study, miR-144-3p in amnion was shown to play an essential role in both term and preterm birth by modulating c-fos and COX2 [41]. In the present study, we demonstrated that COX2 expression and PGE2 release could be increased by upregulation of miR-132-3p. COX2 is the rate-limiting enzyme of prostaglandins [42], and prostaglandins, particularly PGE2 and PGF2α, are mainly derived from amnion and decidua/myometrium, respectively [43,44], which can trigger the rupture of fetal membranes, cervix ripening, and myometrium contraction [45,46]. Korotkov et al. [47] reported that upregulated expression of miR-132-3p could suppress the level of pro-epileptogenic factors, such as COX2 in human cultured astrocytes. Upregulation of extracellular vesicles-packaged miR-132-3p released from CS-treated Th17 cells was shown to induce osteocastogenesis via COX2 downregulation [48]. Recently, administering LNA-anti-miR-132 in mice has been shown to attenuate CCL_4_-induced inflammatory mediators IL-1β and COX2 to alleviate liver fibrosis [49]. These reports indicate that the regulation of COX2 by miR-132-3p appears to be dependent on cell context, which can be either suppressive or promotive. In this study, we also demonstrated that intraperitoneal injection of pregnant mice with miR-132-3p agomir not only caused preterm birth, but also increased the abundance of COX2, suggesting that proinflammatory effects of miR-132-3p in the amnion membranes is an important route to initiate labor.

Additionally, we identified that *DUSP9* is a putative target gene of miR-132-3p by TargetScan and miRDB software and verified by dual luciferase reporter assay. *DUSP9* knockdown was shown to offset the biological effects of miR-132-3p inhibitor on WISH cells. DUSP9 is a member of the largest group of protein phosphatases [50]. Phosphatases are important switches for controlling intracellular signal transduction by regulating the steady-state activities of mitogen-activated protein (MAP) kinases [51,52,53], and they perform important functions in parturition. Lei et al. [54] reported that the anti-inflammatory effect of progesterone is mediated through GR-dependent induction of DUSP1 to inhibit AP-1 signaling, thereby maintaining uterine quiescence during pregnancy. Additionally, the decrease of src-homology phosphatase type-1 (SHP-1) was reported to promote uterine remodeling and plasticity through activation of focal adhesion kinase (FAK) and focal adhesion pathways, facilitating myometrium contraction and leading to labor [55]. Note that DUSP9 was obviously expressed in villous trophoblast, along with its downregulation in placenta across human gestation and in severe preeclampsia [28]. Interestingly, we found that DUSP9 could express in amnion epithelial cells, with a decrease in its abundance at 18.5 dpc versus 15.5 dpc. *DUSP9* has been reported to be involved in the development of various diseases through multi-pathways. In gastric cancer, the low expression of DUSP9 promoted the proliferation of gastric cancer cells by activating the JNK signaling pathway [56]. In hepatocellular carcinoma, decreased DUSP9 was reported to promote cell proliferation through the ERK1/2 pathway, which was associated with poor prognosis [57]. Emanuelli et al. [58] showed that DUSP9 exerted a protective effect against stress-induced insulin resistance by inhibiting ERK and JNK phosphorylation and, to a lesser extent, p38 MAPK phosphorylation. The MAPK pathway mediated by p38 and JNK was shown to play an important role in regulating cell functions, including immune inflammatory response [59]. Previous studies have found the involvement of p38 an JNK in the pathogenesis of inflammation-induced preterm birth [60,61]. P38 MAPK not only participated in functional progesterone withdrawal, but also mediated oxidative stress (OS)-induced senescence in amnion epithelial cells, which contributed jointly to labor initiation [62,63,64]. In the present work, by using p38 inhibitor SB203580 and JNK inhibitor SP600125, we identified silencing *DUSP9* mainly activates p38 and JNK signaling pathways, thus triggering an inflammatory and hormonal cascade. Injection of pregnant mice with miR-132-3p agomir was found to decrease the DUSP9 protein level, and increase the phosphorylation of p38 and JNK protein levels in amnion membranes, suggesting the therapeutic potential of JNK or p38 pathways for preterm labor. Interestingly, *DUSP9* knockout was reported to increase high-fat diet-induced liver inflammatory responses by activating the ASK1-JNK/p38 signaling pathway [25]. Jiang, et al. [65] also demonstrated that DUSP9 acts a suppressor for the development of cardiac hypertrophy through the interaction and dephosphorylation of ASK1 to repress p38 and JNK signaling pathways. By activating the p38 and JNK pathways, *ASK1* could facilitate infection-induced uterine inflammation leading to preterm birth [66]. Therefore, we speculate that DUSP9 may bind to and dephosphorylate ASK1 to participate in the inflammatory response and PGE2 production medicated by the p38 and JNK signaling pathways in the process of labor. 

**Figure 8:**
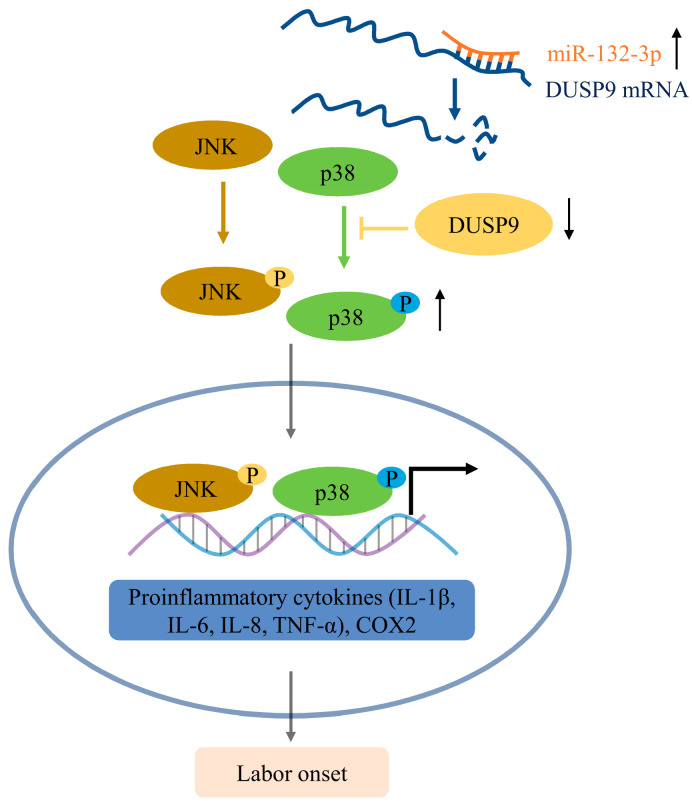
Schematic diagram for the mechanism of miR-132-3p in activating p38 and JNK signaling pathways by downregulating DUSP9, thus promoting inflammation and PGE2 production for parturition initiation, corresponding to the increased expression of IL-1β, IL-6, IL-8, TNF-α, and COX2 in amnion epithelial cells.

In conclusion, we have demonstrated that upregulation of miR-132-3p could suppress the DUSP9 level in amnion to activation of p38 and JNK signaling pathways, thereby inducing inflammatory responses and COX2 as well as PGE2 to trigger parturition (Figure 8). However, due to limitations without human fetal tissues during pregnancy and labor in our study, the correlation between miR-132-3p and *DUSP9* needs to be further elucidated. Understanding the mechanism for triggering labor at term birth may provide an effective strategy to prevent preterm birth and a diagnostic target for preterm labor. 

## 4. Materials and Methods

### 4.1. Animals

Six- to eight-week-old Kunming mice were obtained from the Experimental Animal Center of Huazhong Agricultural University (Wuhan, China) and allowed to adapt to the environment for one week before experiments. All mice were housed at 26 °C constant temperature and 60% relative humidity with a 12 h light/12 h dark cycle and free access to food and water. Female mice mated with male mice overnight from 18:00 p.m. onward. When vaginal plugs were found at 6:00 a.m. the following day, it was defined as gestational day 0.5. The pregnant mice were divided into four groups (with 6–8 mice in each group): 15.5, 16.5, 17.5 and 18.5 days of gestation, respectively. To analyze the gestational changes of miR-132-3p expression, amnion membrane tissues were collected separately from timed pregnant mice at 6:00 a.m. on day 15.5, 16.5, 17.5 and 18.5 post coitum (dpc). The mice amnion membranes collected on day 15.5 and 18.5 of gestation were stored at 4% paraformaldehyde for subsequent immunohistochemical staining analysis.

### 4.2. miRNA Agomir Injection

Agomir NC and miR-132-3p agomir were synthesized by the GenePharma Company (Suzou, China) and chemically modified with a 3′-cholesterol conjugation on the passenger strand to promote its uptake in vivo. To explore whether the injection of miR-132-3p can induce preterm birth, mice on day 15.5 of pregnancy were intraperitoneally injected separately with agomir NC (5 mg/kg BW) and miR-132-3p agomir (5 mg/kg BW) dissolved in 200 μL of phosphate-buffered solution (PBS). For the injected mice, some were allowed to give birth to record the labor time, and some were sacrificed to collect amnion membranes for detecting the abundance of COX2, DUSP9, p38 and JNK as well as phosphorylated p38 and JNK levels. When the miR-132-3p agomir-injected mice delivered the first pup, amnion membranes were collected simultaneously from both the experimental and control groups at the corresponding time points [55]. The collected amnion membranes were flash frozen in liquid nitrogen and stored at −80 °C for further treatment.

### 4.3. Immunohistochemistry

The mice amnion membranes from day 15.5 and 18.5 of gestation were fixed in 4% paraformaldehyde and embedded in paraffin, followed by deparaffinization and rehydration of 5 μm sections. After trypsin-induced antigen retrieval, the sections were incubated in 0.3% hydrogen peroxide for 25 min to quench endogenous peroxidase activity, followed by blocking with 3% Bovine Serum Albumin (BSA) for 30 min at room temperature, and incubation overnight at 4 °C with DUSP9 specific primary antibody (1:50, 503839, ZENBIO, Chengdu, China). Next, the sections were incubated with horseradish peroxidase (HRP) conjugated anti-rabbit secondary antibody (1:1000, ab6721, Abcam, Cambridge, MA, USA) for 50 min, followed by staining with diaminobenzidine for visualization, and then counterstaining with hematoxylin. Digital images were taken by using an Olympus BX53 microscope (BX53, Olympus, Tokyo, Japan).

### 4.4. Reverse Transcription Quantitative Polymerase Chain Reaction (RT-qPCR)

The total RNA of tissues was extracted by homogenization in RNAiso Plus (#9109, Takara, Japan), and the total RNA of cells was extracted using the HP Total RNA Kit (Omega, Doraville, GA, USA). The expression level of miR-132-3p was quantified using the stem-loop RT-qPCR method, and cDNA was synthesized using RevertAid First Strand cDNA Synthesis Kit (#K1622, Thermo Fisher Scientific, Waltham, MA, USA). qPCR was performed using iTaq Universal SYBR^®^ Green Supermix (#1725124, Bio-Rad, Hercules, CA, USA) on CFX384 Touch qPCR system (Bio-Rad, Hercules, CA, USA) at 95 °C for 5 min, 40 cycles of 95 °C for 20 s, 58 °C for 20 s, and 70 °C for 20 s. The expression level of miRNA was normalized to U6, and β-actin was used as an internal control for mRNA. The comparative 2^−ΔΔCT^ method was used to analyze the gene expression levels. The primers used in this study are listed in Table 1.

### 4.5. Western Blot Analysis

Briefly, protein lysates were prepared using an ice-cold RIPA Lysis Buffer (P0013B, Beyotime, Shanghai, China) containing 1% Protease Inhibitor Cocktail (B14001, Bimake, Shanghai, China), 1% Phosphatase Inhibitor Cocktail A (B15001, Bimake, Shanghai, China), and 1% Phosphatase Inhibitor Cocktail B (B15001, Bimake, Shanghai, China). After separation by 10% sodium dodecylsulfate-polyacrylamide gel electrophoresis of Easy^TM^ One-Step PAGE Gel Fast Preparation Kit (PG212, EpiZyme, Shanghai, China), 25 μg of protein extracts were transferred onto 0.22 μm polyvinylidene fluoride (PVDF) membranes (#1620177, Bio-Rad, Hercules, CA, USA). After blocking with 5% non-fat dried milk or 5% BSA in tris-buffered saline Tween (TBST) for 1.5 or 2 h, the PVDF membranes were incubated overnight at 4 °C with specific primary polyclonal antibodies (COX2 (1:2500, PAB31107, Bioswamp, Wuhan, China), phosphorylated JNK at Thr183/Tyr185 (1:1000, 4668, Cell Signaling, Danvers, MA, USA), total JNK (1:1000, 9252, Cell Signaling, Danvers, MA, USA), phosphorylated p38 at Thr180/Tyr182 (1:1000, 4511, Cell Signaling, Danvers, MA, USA), total p38 (1:1000, 8690, Cell Signaling, Danvers, MA, USA), DUSP9 (1:1500, 503839, ZENBIO, Chengdu, China), and β-actin (1:10000, AC004, Abclonal, Wuhan, China) and then with appropriate horseradish peroxidase-conjugated secondary antibodies for 1.5 h. Finally, the immunoreactive protein bands were visualized by Clarity™ Western ECL Substrate (#170-5061, Bio-Rad, Hercules, CA, USA) on ImageQuant LAS 4000 mini System (GE Healthcare, Chicago, IL, USA). β-actin was used as the internal loading control. The integrated density of protein bands was quantified by Image J software.

### 4.6. Cell Culture and Transfection

The WISH cell line was purchased from the Chinese Academy of Sciences bank (Shanghai, China) and cultured at 37 °C in a humidified atmosphere of 5% CO_2_ in DMEM (HyClone, Logan, UT, USA) with 10% fetal bovine serum (Gibco, Carlsbad, CA, USA). To study the effects of miR-132-3p on the levels of interieukin-1β (IL-1β), interieukin-6 (IL-6), interieukin-8 (IL-8), tumor necrosis factor-α (TNF-α), and COX2 as well as PGE2 release, the cells were transfected for 48 h with mimic NC, miR-132-3p mimic, inhibitor NC, and miR-132-3p inhibitor (GenePharma, Suzou, China) using Lipofectamine 2000 reagents (11668-019, Invitrogen, Carlsbad, CA, USA). To determine the effects of si-*DUSP9* on the expression levels of IL-1β, IL-6, IL-8, TNF-α, and COX2, as well as PGE2 production, the cells were transfected for 48 h with NC and si-*DUSP9* using Lipofectamine^®^ RNAiMAX Reagent (13778-150, Invitrogen, Carlsbad, CA, USA). To verify the involvement of p38 and JNK signaling pathways in the induction of IL-1β, IL-6, IL-8, TNF-α, and COX2 as well as PGE2 secretion by *DUSP9* siRNA, the cells were transfected with NC or si-*DUSP9* for 24 h, followed by 24 h of treatment with dimethyl sulfoxide (DMSO), p38 inhibitor SB203580 (10 μM, Selleck, Houston, TX, USA), or JNK inhibitor SP600125 (20 μM, Selleck, Houston, TX, USA). The synthesized sRNAs sequences are shown below: negative control (NC): 5′-UUCUUCGAACGUGUCACGUTT-3′ and 5′-ACGUGACACGUUCGGAGAATT-3′; miR-132-3p mimic: 5′-UAACAGUCUACAGCCAUGGUCG-3′ and 5′- ACCAUGGCUGUAGACUGUUAUU-3′; inhibitor NC: 5′-CAGUACUUUUGUGUAGUACAA-3′; miR-132-3p inhibitor: 5′-CGACCAUGGCUGUAGACUGUUA-3′; si-*DUSP9*-1: 5′-AGGGAGGCUUCAGCAGAUUTT and 5′-AAUCUGCUGAAGCCUCCUTT; si-*DUSP9*-2: 5′-CUCUCAACGAUGCCUAUGATT-3′ and 5′-UCAUAGGCAUCGUUGAGAGTT-3′; si-*DUSP9*-3: 5′-GUCCUAAUCAACGUGCCUATT-3′ and 5′-UAGGCACGUUGAUUAGGACTT-3′.

### 4.7. Magnetic Luminex^®^ Assay

To quantify the concentrations of IL-1β, IL-6, IL-8, and TNF-α, cell-free supernatants were analyzed using Human Magnetic Luminex^®^ Assays (LXSAHM, R&D Systems, Minneapolis, MN, USA) on the Luminex 200 System as instructed by the manufacturer.

### 4.8. Enzyme-Linked Immunosorbent Assay

Briefly, cultured cell supernatant samples were collected from different groups and centrifuged at 3000 g for 5 min to pellet cell debris. The concentration of PGE2 was determined using enzyme-linked immunosorbent assay (ELISA) kit (HM10090, Bioswamp, Wuhan, China) according to the manufacturer’ instructions. The optical density (OD) was measured with the EnSpire plate reader (PerkinElmer) at 450 nm. 

### 4.9. Dual-Luciferase Reporter Assay

Online analysis websites TargetScan (www.targetscan.org/vert_71/, accessed on 8 January 2022) and miRDB (mirdb.org, accessed on 8 January 2022) were used to predict the target genes of miR-132-3p and putative binding site between miR-132-3p and *DUSP9* 3′untranslated region (3′UTR). The *DUSP9* 3′UTR containing the seed sequence binding site of miR-132-3p was amplified by PCR, followed by digesting the PCR product and pmirGLO vector using *MssI* (FD1344, Thermo Fisher Scientific, Waltham, MA, USA) and *XhoI* (FD0694, Thermo Fisher Scientific, Waltham, MA, USA). The fragment of *DUSP9* 3′UTR was inserted into the pmirGLO vector using T4 DNA ligase (M0202S, NEB). The pmirGLO-*DUSP9*-3′UTR-MUT vector was constructed using site-directed mutagenesis. WISH cells were transfected with miR-132-3p mimic or NC and the three reported plasmids. After 48 h of transfection, cells were lysed to collect the supernatant. The luciferase activity was measured by Dual-luciferase^®^ Reporter System (E1980, Promega Corporation, Madison, WI, USA) according to the manufacturer’s protocol, and relative luciferase activity was calculated as the ratio of the firefly luciferase activity to Renilla luciferase activity.

### 4.10. Statistical Analysis

In this study, all data were presented as means ± standard error of means (SEM) and were graphed using GraphPad Prism software. Unpaired Student’s test was performed for comparison between two groups. Comparisons among different groups were conducted by one-way ANOVA. * *p* < 0.05, ** *p* < 0.01, # *p* < 0.05, ## *p* < 0.01 indicated statistical significance for different groups or experiments.

## Figures and Tables

**Table 1:** Primer sequences for RT-qPCR.

Gene	Primer Sequences (5′–3′)	Size (bp)
*U6*	F: CTCGCTTCGGCAGCACA R: AACGCTTCACGAATTTGCGT	94
*miR-132-3p*	F: ACACTCCAGCTGGGTAACAGTCTACAGCC R: CTCAACTGGTCTCGTGGA	72
*β-actin* (human)	F: AGCGGGAAATCGTGCGTG R: CAGGGTACATGGTGGTGCC	309
*DUSP9* (human)	F: GCTACCTGGCCTACTACCTCC R: CATCAGAGCAGTCGGAGCCC	171
*IL-1β* (human)	F: AGCTACGAATCTCCGACCAC R: GCCTCGTTATCCCATGTGTC	190
*IL-6* (human)	F: AGTAGTGAGGAACAAGCCAGAG R: TTGGGTCAGGGGTGGTTATTG	106
*IL-8* (human)	F: TACTCCAAACCTTTCCACCCC R: CAACCCTCTGCACCCAGTTT	148
*TNF-α* (human)	F: ATCTTCTCGAACCCCGAGTGA R: GAGTAGATGAGGTACAGGCCC	171
*COX2* (human)	F: CTGCGCCTTTTCAAGGATGG R: CCCCACAGCAAACCGTAGAT	135
*β-actin* (mouse)	F: CACGATGGAGGGGCCGGACTCATC R: TAAAGACCTCTATGCCAACACAGT	241
*DUSP9* (mouse)	F: AATGTCACCCCCAACCTTCC R: CCCACAGTTCTGCGACAAGG	153
*IL-1β* (mouse)	F: ATGAAAGACGGCACACCCAC R: TACCAGTTGGGGAACTCTGC	146
*IL-6* (mouse)	F: TCACAGAGGATACCACTCCCA R: GCAAGTGCATCATCGTTGTTC	148
*IL-8* (mouse)	F: CTAGGCATCTTCGTCCGTCC R: TTCACCCATGGAGCATCAGG	200
*COX2* (mouse)	F: CCTGGAACATGGACTCACTCA R: TGTGTACGGCTTCAGGGAGA	187

## Data Availability

The data presented in this study are available on request from the corresponding author.
